# Accelerometer- and Gyroscope-Based Sensor System for Detecting Swallowing, Speaking, and Coughing Events in ICU Patients

**DOI:** 10.3390/s26217089

**Published:** 2026-11-09

**Authors:** Constantijn Coppers, Stijn Van de Velde, Guylian Stevens, Jonas Van Der Donckt, Marija Stojchevska, Sofie Van Hoecke, Pascal Verdonck

**Affiliations:** 1 Department of Electronics and Information Systems—IBiTech, Ghent University, Korneel Heymanslaan, 9000 Ghent, Belgium; constantijn.coppers@ugent.be; 2 Partnership of Anesthesia, vzw Maria Middelares, Buitenring Sint-Denijs 30, 9000 Ghent, Belgium; stijn.vandevelde@mijnziekenhuis.be; 3 Department CTO, vzw Maria Middelares, Buitenring Sint-Denijs 30, 9000 Ghent, Belgium; jonasvanderdonckt@gmail.com (J.V.D.D.); marija.stojchevska@ugent.be (M.S.); sofie.vanhoecke@ugent.be (S.V.H.); 4 IDLab, Department of Electronics and Information Systems, Ghent University—Imec, Technologiepark-Zwijnaarde 122, 9052 Zwijnaarde, Belgium; pascal.verdonck@ugent.be

**Keywords:** inertial measurement unit, swallowing detection, cough detection, speech detection, intensive care unit

## Abstract

(1) Background: Swallowing, coughing, and speaking are clinically relevant physiological events, but they are not routinely and continuously monitored in intensive care. This study evaluates the feasibility of detecting these events using inertial measurement unit (IMU) signals acquired from the cricoid and thoracic regions. (2) Methods: Data were collected from 29 patients in the intensive care unit (ICU) during predefined swallowing, coughing, speaking, and movement tasks. A classical machine learning pipeline was developed and evaluated using leave-one-patient-out cross-validation. (3) Results: Cricoid and thoracic accelerometer signals were informative for event detection, with the thoracic sensor particularly contributing to cough detection. Speaking ($F_{1}=0.80$), coughing ($F_{1}=0.72$), and aggregated swallowing ($F_{1}=0.74$) were detected in this controlled experimental setting. (4) Conclusions: The results support the feasibility of IMU-based detection of prompted swallowing, coughing, and speaking events in ICU patients. Further validation under spontaneous and clinically heterogeneous conditions is required before continuous clinical monitoring can be assessed.

## 1. Introduction

Swallowing, coughing, and breathing are vital physiological processes that are closely interconnected. Their frequency and temporal characteristics may provide clinically relevant information. Continuous or long-term monitoring of these events could potentially provide additional information about patient status and longitudinal changes, but the present study does not evaluate spontaneous events or clinical outcomes. In this manuscript, pneumonia, chronic obstructive pulmonary disease (COPD), and interstitial lung disease (ILD) are discussed as underlying medical conditions, dysphagia as a swallowing-related symptom or disorder, and intensive care unit (ICU) readmission as a clinical outcome, rather than as biomarkers in themselves [1,2,3,4,5,6,7,8,9,10,11,12,13].

Existing clinical assessments of swallowing and cough are generally episodic and are not designed to provide continuous event monitoring. Swallowing assessment may include a clinical examination and instrumental methods such as a videofluoroscopic swallowing study (VFSS) and fiberoptic endoscopic evaluation of swallowing (FEES), depending on the clinical question [14,15,16]. Cough assessment includes both patient-reported measures and objective approaches; acoustic methods are one possible objective approach, but they can be affected by environmental noise and privacy considerations [17,18]. Accordingly, in this study we use the term reference clinical methods rather than gold-standard methods when describing established swallowing and cough assessments. The state of the art in respiratory monitoring includes multiple sensor-based and contactless approaches, but the present work does not evaluate respiratory rate estimation and therefore does not make a quantitative claim about respiratory monitoring [19].

**Figure 1:**
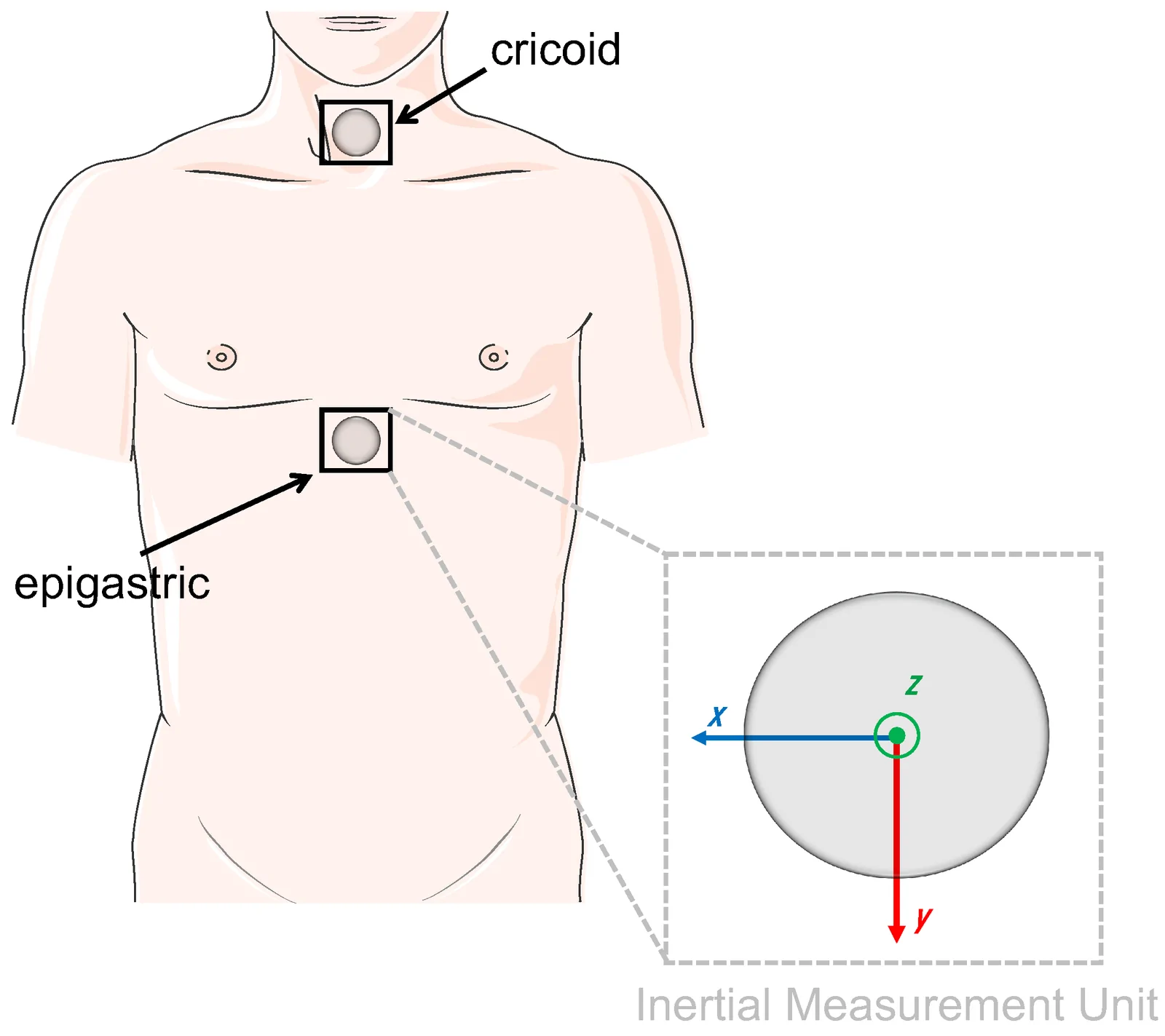
Illustration of the sensor placement. Two IMUs, each capturing tri-axial accelerometer and gyroscope signals, are attached to the body: one is positioned on the cricoid cartilage, and the other is placed on the epigastric region of the thorax.

To address the need for unobtrusive event monitoring, this study uses two inertial measurement units (IMUs): one placed at the cricoid region and one placed at the thoracic/epigastric region (Figure 1). The sensors capture tri-axial accelerometer and gyroscope signals associated with physiological and body movements.

The aim of this study is to determine whether these IMU signals can distinguish prompted swallowing, coughing, and speaking events in ICU patients and to quantify how sensor location and signal modality affect classification performance. The primary research questions are as follows: (1) How accurately can the proposed system detect swallowing, coughing, and speaking events in a patient-independent evaluation? (2) Does combining cricoid and thoracic signals improve detection compared with cricoid signals alone? (3) What is the additional contribution of gyroscope signals compared with accelerometer signals? Because wet and dry swallowing were both recorded, we additionally examine whether these two swallowing conditions can be distinguished.

## 2. Materials and Methods

The study was conducted in accordance with the Declaration of Helsinki, and the protocol was approved by the Ethics Committee of Maria Middelares (Belg. Regnr. B0172024000002 on 24 April 2024).

### 2.1. Data Collection

Data were collected from 29 patients admitted to the ICU of AZ Maria Middelares (Ghent, Belgium) from June 2024 to March 2025. Two inertial measurement units (IMUs; Movesense MD, Movesense, Vaanta, Finnland) were placed on each patient, one at the cricoid region and one at the thoracic/epigastric region, capturing tri-axial accelerometer and gyroscope signals at 208 Hz, yielding twelve raw signal channels per recording (Figure 1). The sensors were wirelessly connected to a custom-built computer dashboard, allowing the principal investigator (PI) to monitor, store, and timestamp the prompted patient actions during the recording.

The ICU population was selected because the longer-term clinical motivation of the SwaReCo concept is monitoring physiological events in critically ill patients; however, the present experiment was designed as a controlled feasibility study rather than as a test of continuous bedside monitoring. Inclusion criteria were as follows: being older than 18 years; being able to give informed consent; being admitted to the ICU; being able to perform voluntary swallowing, coughing, and speaking movements; and being non-ventilated and breathing spontaneously. Exclusion criteria were pregnancy and known allergies to adhesives. Twenty-five participants (86.2%) were admitted following cardiac surgery, and four (13.8%) were admitted for a non-surgical medical condition. Recording time was defined as the timestamp of sensor application. ICU day was calculated using calendar dates, with the date of ICU admission designated as day 1. The exact elapsed time from ICU admission to sensor application was a median of 26.7 h (IQR 20.7–46.8; range 2.0–81.6). The median ICU day at recording was 2 (IQR 2–3; range 1–4). At the time of recording, all 29 participants were awake, clinically appropriate in their responses, and oriented to time and place. The population rationale can be found in Table 1.

Each patient performed up to six recordings, covering four types of actions: swallowing, coughing, speaking, and movement (head, arms and legs). The swallowing category consisted of two separate recordings: one for dry swallowing, referring to the act of swallowing saliva, and one for wet swallowing, involving the ingestion of a small amount (50 mL) of liquid. For both swallowing recordings, patients performed five repetitions. Coughing and speaking were also repeated five times. Patients were instructed to pause approximately ten seconds between repetitions; in practice, most patients performed the repetitions at their own pace and in quicker succession (Section 3.1). Each recording was started manually shortly before the first repetition and stopped shortly after the last. For the speaking recordings, patients were instructed to repeat a standard sentence five times. The movement recordings consisted of two separate recordings: one for a single left turn and another for a single right turn. The recording protocol and the number of usable recordings were not identical for every patient; the resulting counts are reported in Table 2.

### 2.2. Data Preparation

The raw signals from the two Movesense MD sensors were first synchronized using computer-side timestamps assigned upon data reception via Bluetooth. This aligned all signals and annotations to a shared time reference. Subsequently, the signals were resampled from 208 Hz to 200 Hz using linear interpolation.

Annotations were made post hoc by an independent analyst based on timestamps logged during the recording sessions. For each action, the analyst manually defined the start and end times using the logged markers and surrounding signal characteristics. Specifically, the following events were explicitly segmented: *swallowing wet*, *swallowing dry*, *coughing*, and *speaking*. Segments that were ambiguous were conservatively labeled as *artifacts* and excluded from further analysis. All remaining, explicitly unannotated samples were assigned the label *background*.

### 2.3. Preprocessing

To enhance signal quality, all raw time-series signals were filtered using soft wavelet thresholding with the Daubechies 4 wavelet, following the approach of Stevens et al.  [20]. Each signal was decomposed into four levels using the discrete wavelet transform. The noise level was estimated as $\hat{\sigma }=\mathrm{median}(|d_{1}\left|\right)/0.6745$, where $d_{1}$ denotes the finest-scale detail coefficients, and the universal threshold $\lambda =\hat{\sigma }\sqrt{2\ln N}$, with *N* being the number of samples in the full recording, was applied by soft thresholding to the detail coefficients of all four levels  [21]. The approximation coefficients were left unchanged, and the signal was reconstructed by the inverse discrete wavelet transform. Wavelet denoising was implemented in PyWavelets (version 1.8.0). For the cricoid tri-axial accelerometer signals, additional high-pass filtering was applied to remove low-frequency body motion and the static gravity component. Specifically, the high-pass filtered signal $x_{\mathrm{HP}}$ was obtained by subtracting a low-pass version $x_{\mathrm{LP}}$ from the original signal *x*, as defined by $x_{\mathrm{HP}}=x-x_{\mathrm{LP}}$. The low-pass version was derived using a Gaussian filter with a standard deviation of σ=0.20 s. Furthermore, the tri-axial gyroscope signals from the cricoid sensor were wavelet-filtered only. For the thorax sensor, the Euclidean norm of the accelerometer and gyroscope signals was computed to reduce dimensionality and emphasize the overall motion magnitude.

### 2.4. Feature Extraction

To convert continuous time-series signals into structured input for classification, a sliding-window approach was used. Each signal was segmented into overlapping windows of 1.0 s (200 samples) with a stride of 0.05 s (10 samples). For each window, a majority-voting scheme assigned a class label based on the most frequent ground-truth label among its constituent time samples. In this manuscript, a *sample* refers to one original time point, a *window* refers to one 1.0 s input segment, and an *event* refers to one contiguous annotated segment of a target class. The sample-based classification metrics are calculated on reconstructed time-point predictions after the window-level predictions have been mapped back to the signal timeline; these metrics therefore do not represent independent windows.

Next, feature vectors were computed for each individual sensor channel and concatenated into a single feature vector per window. Before feature selection, the exact number of attributes depended on the signal configuration and processing stage; the final numbers used in each configuration are reported explicitly in Table 3.

Features were extracted using the tsfresh Python 3.1 package [22]. Before model training, a coarse relevance filter was applied once to remove attributes without relevance to the target classes: in six random subsampling iterations, the tsfresh relevance test was applied to the data of seven patients randomly selected without replacement, and an attribute was retained if it was selected in at least four of the six iterations. Because the subsets were drawn from all patients, the held-out patient of a cross-validation fold could have contributed to this filter. This influence is expected to be minimal: the filter only removed attributes that were consistently irrelevant, and a large number of attributes was retained (3298 of the 6264 extracted attributes for the full sensor configuration; Table 3). The actual selection of informative features was left to the CatBoost classifier, which performs implicit feature selection during training and was trained within each fold on the training patients only. To assess the influence of feature preselection, we repeated the analysis with a fixed set of 22 predefined features, computed per window. The set comprises 13 statistical features, 2 complexity features, and 7 frequency-domain features (dominant frequency, spectral entropy, and band energy in five bands), and it was defined without reference to the study data. The same cross-validation scheme and models were used as in the main analysis. The full feature set is given in Supplementary Table S1.

### 2.5. Model Development

A supervised learning approach was used to train a classifier that outputs a probability vector indicating the likelihood of belonging to each of five target classes: background, coughing, speaking, swallowing wet, and swallowing dry. A CatBoost classifier was selected due to its strong performance on structured, high-dimensional data, its ability to model non-linear relationships, and its good out-of-the-box performance [23,24].

The model is based on gradient-boosted decision trees and performs implicit feature selection during training. As such, no prior extensive feature selection procedure is needed, since the model automatically selects from the most relevant features. It was configured with fixed hyperparameters that were set in advance and not tuned on the data: 500 iterations, a learning rate of 0.03, a tree depth of 6, and the cross-entropy loss function. To address class imbalance, class weights were set to balanced.

### 2.6. Post-Processing

To obtain temporally localized event predictions from the classifier output, a post-processing pipeline was applied. For each windowed input, the classifier yields a probability distribution over the target classes. These probabilities are assigned to the central time point of their corresponding window and spread symmetrically across a time interval equal to the stride. This produces a continuous per-sample probability distribution.

Following this, the probability distribution is smoothed using a Gaussian filter with a standard deviation of σ=0.1 s to reduce short-term fluctuations. Finally, each time sample is assigned the class with the highest smoothed probability, resulting in a discrete sequence of predicted event labels.

### 2.7. Experiment Configuration

To evaluate the impact of different sensor modalities on classification performance, four sensor configurations were defined. In each configuration, a modality was considered included if its extracted features were part of the concatenated feature vector used for model training and evaluation; otherwise, it was excluded.

An overview of the included signals per configuration is provided in Table 4. The CA configuration includes only the tri-axial accelerometer data from the cricoid sensor. CA+CG adds the tri-axial cricoid gyroscope signals. CA+TA includes the accelerometer signals from both the cricoid and thorax sensors, with the thorax data represented by signal norms. Finally, CA+CG+TA+TG combines all available signals, including the gyroscope signals from the thorax.

### 2.8. Model Evaluation

All models were evaluated using leave-one-patient-out cross-validation (LOPOCV) across 29 patients, resulting in 29 folds. In each fold, data from one patient were withheld for evaluation, and the classifier was trained on the remaining patients only. The coarse relevance filter described in Section 2.4 was applied once before cross-validation.

Sample-based evaluation

Predictions were reconstructed at individual time points before calculating sample-based metrics. For each class, recall, precision, and $F_{1}$ score were calculated on the held-out folds, and the scores were then averaged across folds. A single confusion matrix was constructed by concatenating predictions from all test folds and row-normalizing it so that diagonal values represent per-class recall.

Event-based evaluation

To assess the detection of complete annotated events, we used an event-overlap measure. For each ground-truth event, the proportion of its duration overlapping with a predicted segment of the same class was counted as a true-positive fraction; the remaining duration was treated as a false-negative fraction. This duration-overlap formulation is not equivalent to a conventional event-detection metric based on discrete event-level true positives, false positives, and onset/offset tolerances. It is retained here for consistency with the original analysis, and this limitation is considered in the Discussion Section.

Signal importance

To assess the relative importance of each signal source, the feature importance scores from the CatBoost classifier, using the PredictionValuesChange method, were leveraged. This method estimates how much the model’s predictions change when a given feature is varied.

The process was as follows:

1. For each fold, the trained model’s feature importances were extracted.
2. These were concatenated across all 29 folds.
3. Features were grouped by their originating signal source.
4. The average (and standard deviation) of the importance value was computed per signal.

## 3. Results

### 3.1. Exploratory Data Analysis

Dataset characteristics

A total of 174 recording slots were planned across 29 patients, corresponding to up to six recordings per patient. The number of usable recordings differed by action type; the observed counts are reported in Table 2. In particular, the table contains 28 coughing recordings and 30 wet-swallowing recordings. One patient was unable to perform the coughing, speaking, and wet-swallowing tasks, and another patient was unable to perform the dry-swallowing task, so these recordings are missing. For four patients, the first attempt at a task was not completed, and the task was repeated (wet swallowing in two patients and dry swallowing and speaking in one patient each); both recordings of these patients are included in the dataset. As a result, the table contains 28 coughing and 30 wet-swallowing recordings, and the counts of 29 for dry swallowing and speaking each comprise one missing and one repeated recording. The average recording durations vary by action type: turning left (11.21 s) and turning right (9.48 s) are the shortest, while wet swallowing (30.07 s) and dry swallowing (23.79 s) are the longest. Coughing and speaking recordings fall in between, with average durations of 15.41 s and 16.45 s, respectively (Table 2). Because recordings were started and stopped manually around the task, these durations mainly reflect the time patients needed to complete the five repetitions. The median interval between the onsets of consecutive repetitions was 4.0 s for dry swallowing, 5.1 s for wet swallowing, 2.4 s for speaking, and 1.9 s for coughing, all of which were shorter than the instructed pause of approximately ten seconds.

Segment statistics

Table 5 provides an overview of the number of samples and annotated events per label. Speaking (181 events) and coughing (174 events) are the most frequently annotated actions. Swallowing wet (151 events) and swallowing dry (149 events) are less prevalent. Furthermore, 121 artifacts were manually identified during the annotation process (Section 2.1).

**Figure 2:**
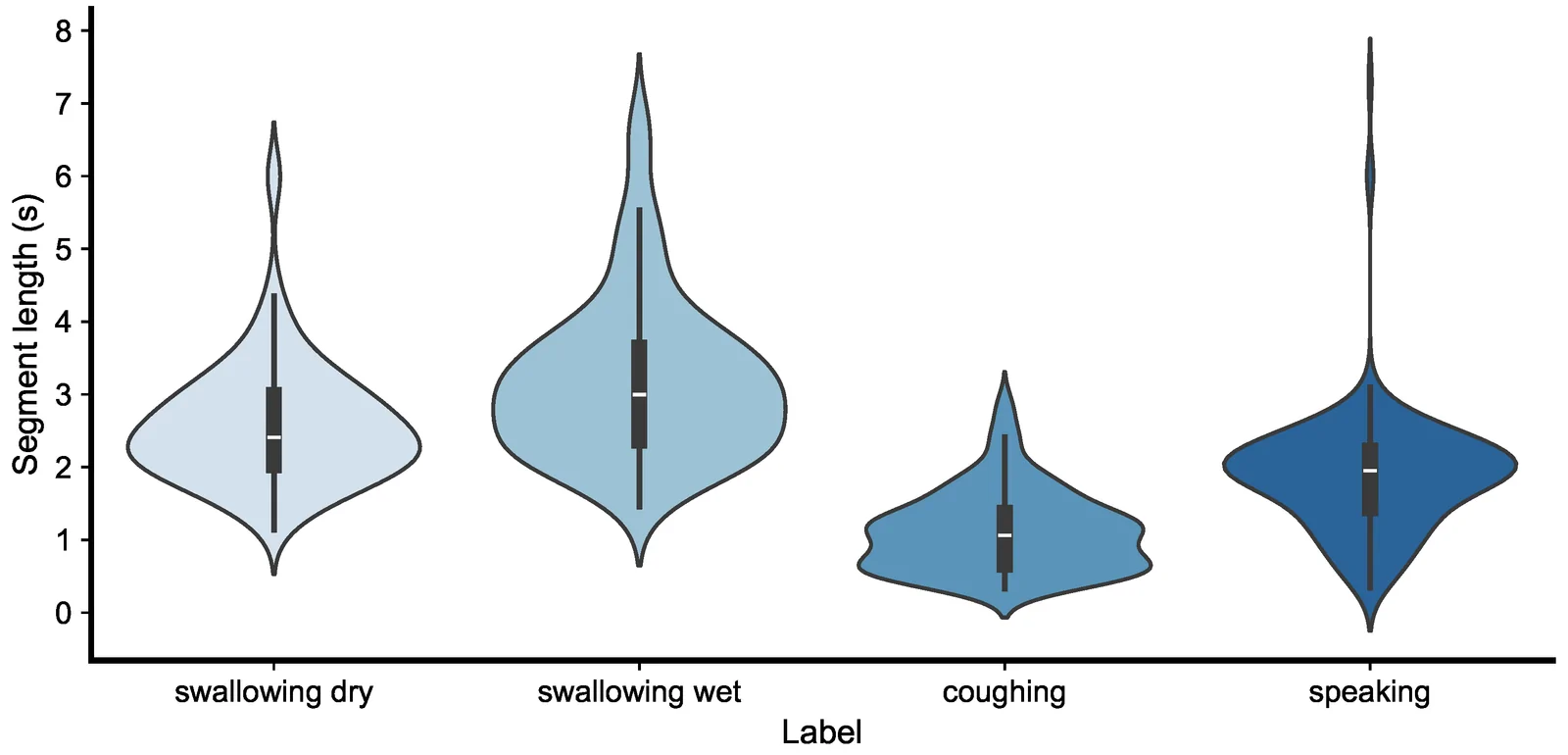
Violin plots of the segment length for each class.

The segment length distributions per class are visualized in Figure 2. Coughing segments are the shortest and most consistent, with a mean of 1.10 s and low variability (0.55 s). Speaking segments exhibit a sharp peak near the mean of 1.88 s and show the widest spread, with a negatively skewed distribution. Swallowing events are generally longer and positively skewed. Wet swallowing has the highest mean duration (3.18 s) and variability (1.10 s), while the duration of dry swallowing events averages 2.57 s.

Extracted features

From each signal channel, tsfresh initially extracted 783 features. The earlier description of 4698 attributes refers to the aggregate pre-filtering representation for the specific signal set used at that processing stage, whereas the 783 figure refers to the number of features generated per individual signal channel. After feature selection, the number of retained features depended on the sensor configuration. Table 3 reports the retained feature counts per signal channel and their total. The apparent discrepancy between 3275 and 3298 in the previous version resulted from mixing intermediate and final feature sets; the final configuration-specific counts in Table 3 should be used when describing the evaluated models.

### 3.2. Confusion Matrices

**Figure 3:**
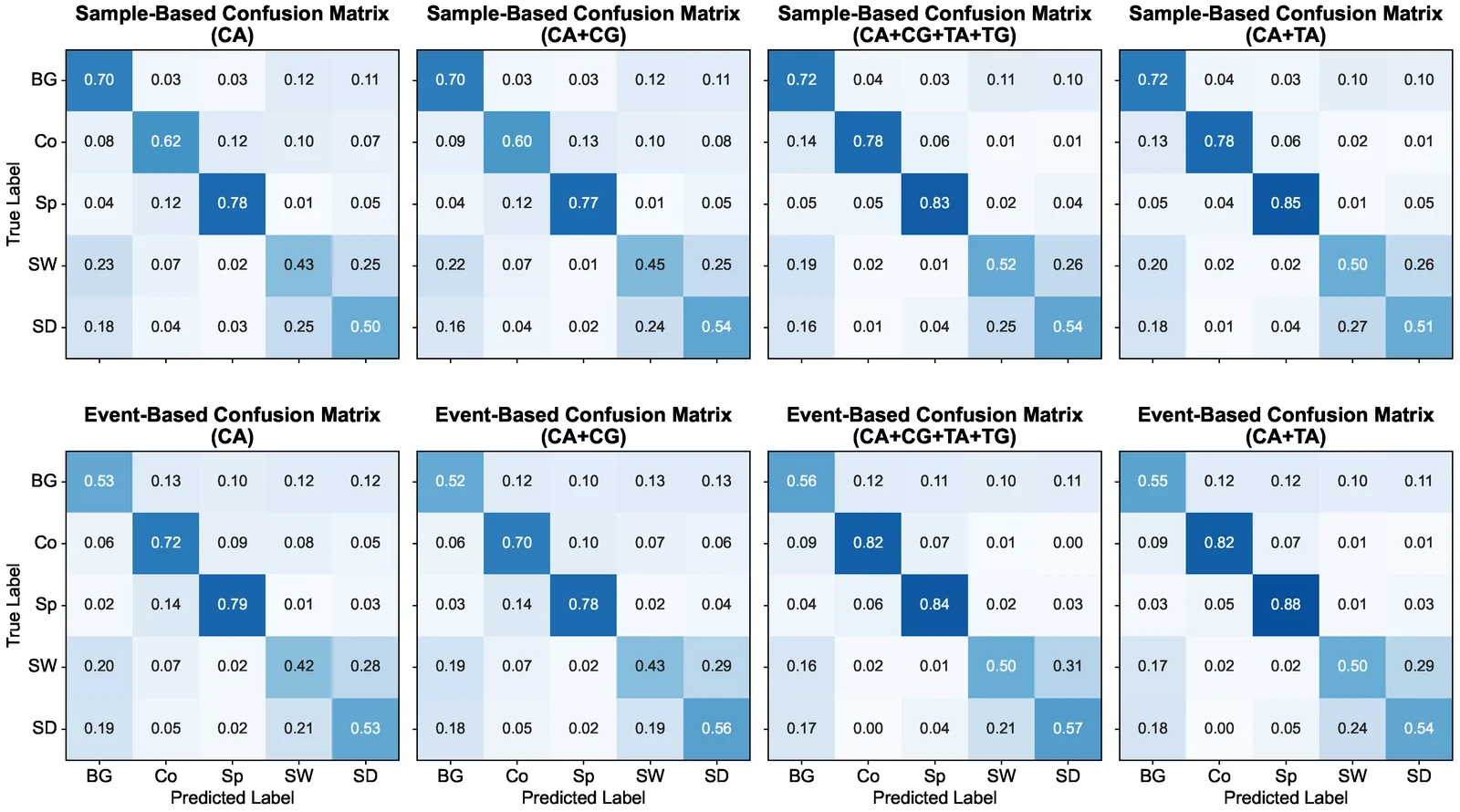
A 2×4 grid of normalized confusion matrices, aggregated over all cross-validation folds. Each column represents a distinct sensor configuration. The rows distinguish among types of confusion matrices. The first row represents the sample-based confusion matrices, while the second row represents the event-based confusion matrices. Abbreviations: background (BG), coughing (Co), speaking (Sp), swallowing wet (SW), swallowing dry (SD); see Table 4 for the sensor configurations.

An overview of the confusion matrices is shown in Figure 3, comparing sample-based and event-based predictions across different sensor configurations.

Overall, three patterns emerge consistently across all modalities:

- Speaking is the most reliably recognized class, showing the highest recall (diagonal elements);
- Swallowing wet and swallowing dry are frequently confused with each other and with the background class;
- The background class shows a marked drop in recall under event-based evaluation.

Comparing sensor configurations, we observed that including the thorax sensors led to improved recognition of coughing (by approx. +0.15) and speaking (up to +0.07) and reduced confusion between them. Minor differences were observed between accelerometer-only and full sensor setups, with accelerometers alone being slightly better at recognizing speaking events.

### 3.3. Per-Class Scores

**Figure 4:**
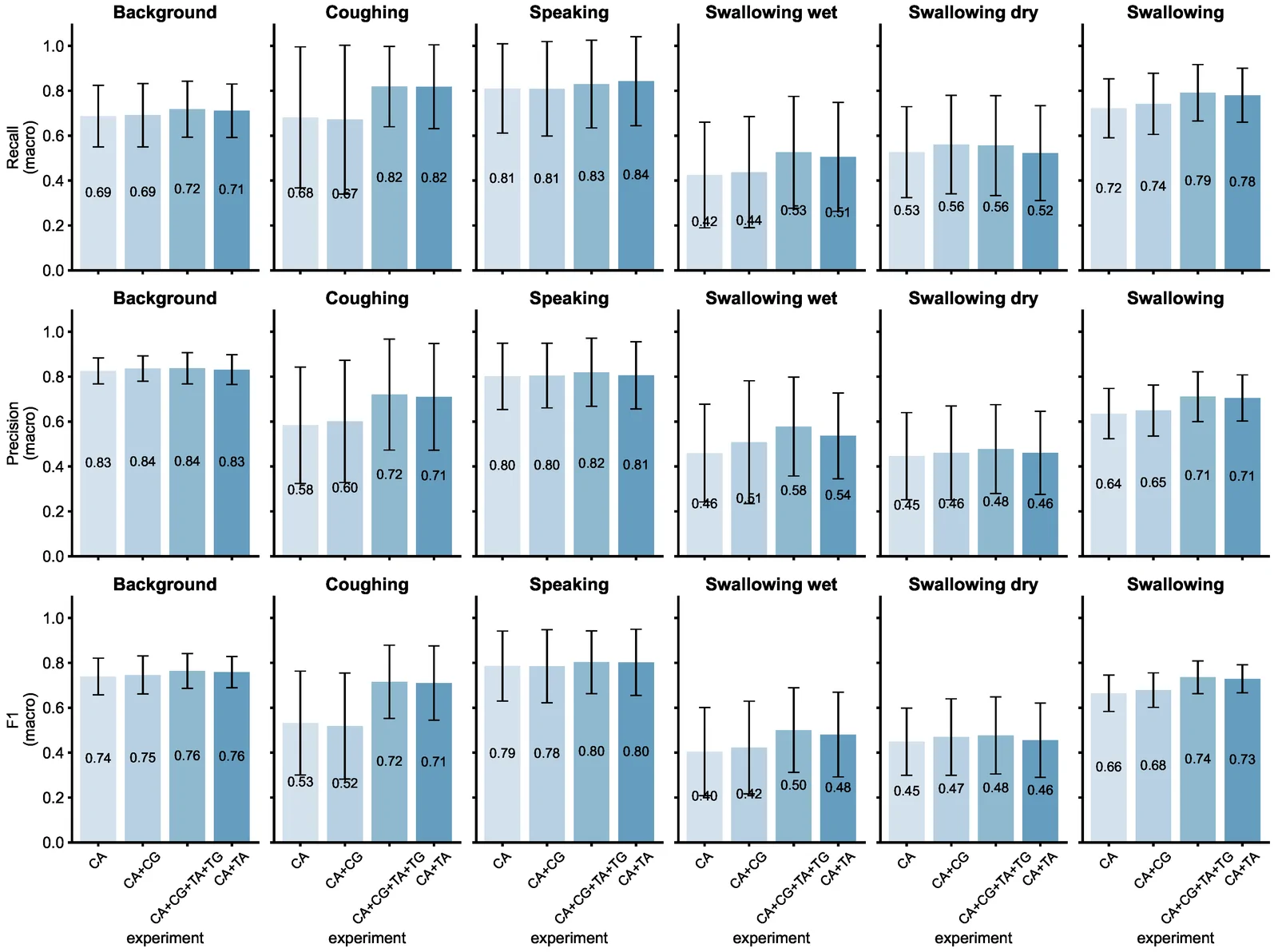
Bar plot displaying the per-class recall, precision, and $F_{1}$ scores, evaluated on a per-sample basis. Each bar is grouped per sensor configuration. All scores are averaged across folds.

Figure 4 shows the class-wise recall, precision, and $F_{1}$ scores across sensor modalities.

The swallowing wet and dry classes are the most challenging to classify, showing the lowest $F_{1}$ scores (typically 0.40–0.50) and high variability. Mean scores are slightly higher when gyroscope signals are added, but these differences are small relative to the inter-patient variability. In contrast, the aggregated swallowing class achieves considerably higher and more stable scores.

Coughing events exhibit lower precision than recall across all modalities. The addition of thorax sensors leads to a marked performance improvement ($F_{1}$ increase of 0.18–0.20).

Speaking consistently shows the highest $F_{1}$ scores (>0.78), with minimal differences across modalities.

The background class yields stable scores, although precision tends to be about 0.12 higher than recall.

Finally, high standard deviations across metrics indicate substantial inter-patient variability, which is evident in all classes.

### 3.4. Feature Importance

Feature importance quantifies the contribution or *weight* of each feature to the model’s predictions. Features with higher importance values exert a stronger influence on the output of the model, while those with lower values contribute less.

**Figure 5:**
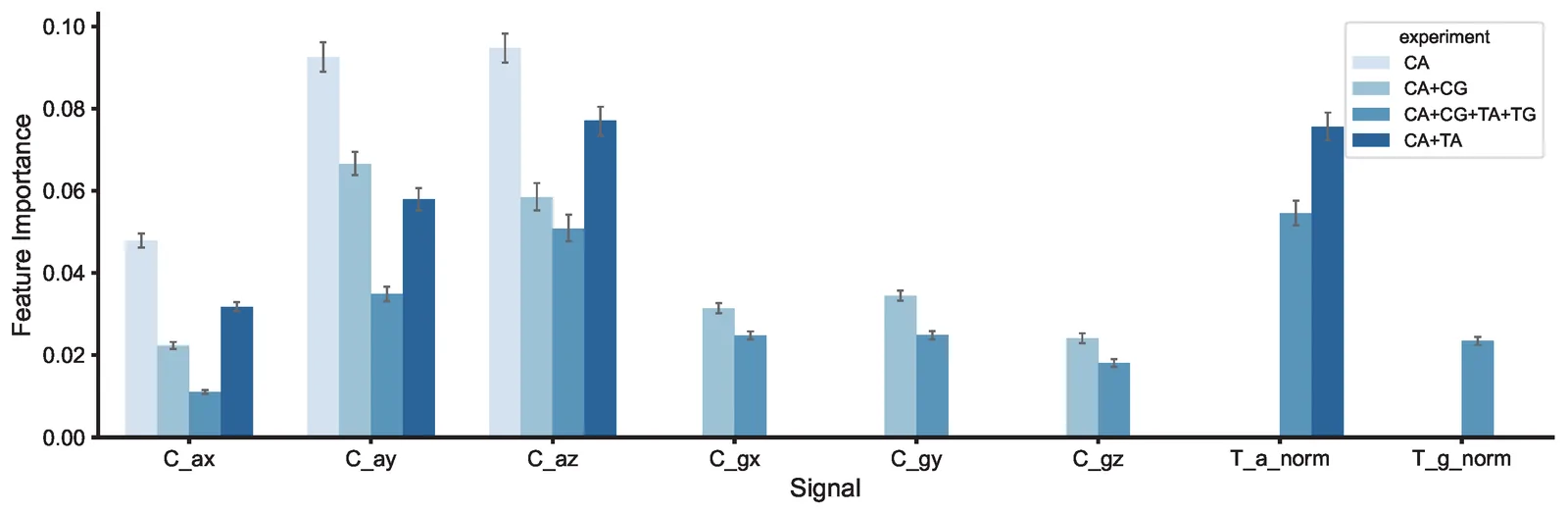
Grouped bar plot of the mean (with error bars) feature importance per signal.

Figure 5 presents a grouped bar plot showing the mean (with error bars) feature importance per signal across four sensor configurations.

This figure demonstrates a clear pattern: accelerometer signals generally have a higher mean importance than gyroscope signals. In particular, the cricoid accelerometer signals along the *y*- and *z*-axes, as well as the thorax norm accelerometer, exhibit the highest average importance. An exception is the cricoid *x*-axis accelerometer, which shows relatively low importance.

## 4. Discussion

### 4.1. Reflection on the Methodology

This study is based on prompted (not *spontaneous*) actions performed under a controlled recording protocol. Consequently, the findings should be interpreted as a preliminary feasibility assessment rather than evidence that the system can continuously monitor patients under routine ICU conditions. The experiment did not include spontaneous swallowing, coughing, or speaking; nursing activities; posture changes; sensor displacement; or other common sources of ICU motion and signal interference. In addition, although patients were instructed to pause approximately ten seconds between repetitions, they performed the repetitions in quicker succession (median onset-to-onset interval of 1.9–5.1 s depending on the action; Section 3.1). For coughing in particular, consecutive events were sometimes less than one window length (1.0 s) apart, such that a single window could contain parts of two events, which complicates the separation of individual events. Conversely, closely spaced repetitions yield a much higher proportion of event samples than continuous monitoring, in which spontaneous events are sparse; performance, and precision in particular, may therefore be lower in that setting. The dataset was also small, and heterogeneous clinical information is insufficiently reported in the current manuscript. Ground-truth annotations were generated post hoc by a single analyst using recorded markers and signal characteristics, which may introduce annotation subjectivity and bias. Furthermore, a single classifier (CatBoost) was evaluated, and differences between sensor configurations are reported descriptively as mean ± standard deviation across patients, without formal statistical testing. Given the small cohort and the high inter-patient variability, these comparisons should be regarded as exploratory; a systematic comparison with other classifiers and a confirmatory evaluation of sensor configurations require a larger cohort.

The tsfresh relevance filter is supervised and was applied once, before cross-validation, on random subsets of seven patients drawn from all 29 patients (Section 2.4). The held-out patient of a fold could therefore have contributed to this filter, so the leave-one-patient-out estimates may be slightly optimistic. This effect was expected to be small, as the filter is a coarse univariate screen that retained about half of the attributes and does not optimize the classifier, while all model fitting was done on the training patients of each fold only. Repeating the filter within each training fold would remove this bias and should be done in future work.

Because feature preselection was performed before cross-validation, the reported performance estimates may be optimistic. A sensitivity analysis using 22 predefined features did not show a meaningful change or showed a small to moderate decrease in performance, which partly addresses this concern but does not eliminate it. Validation on an independent dataset, with feature selection nested within the cross-validation loop, is needed to confirm our findings.

### 4.2. Sensor Modality

Accelerometers proved to be particularly informative for detecting the target events. The cricoid accelerometer effectively captured mechanical vibrations produced during phonation (speech), swallowing, and coughing. The thorax accelerometer notably improved performance for cough detection, likely due to the strong muscular activity in the thoracic region during coughing.

The cricoid gyroscope showed limited additional value relative to accelerometer signals, although some differences were observed for swallowing detection. The present ablation design does not include a thorax-only configuration, so the independent contribution of the thoracic sensor cannot be completely separated from the effects of combining sensor locations.

### 4.3. Generalizability

The model exhibited limited generalizability across patients, with high variability in performance metrics. This can be attributed to a relatively small dataset and a low event frequency per participant (about five repetitions for each action), which restricted the model’s ability to generalize effectively. Additionally, inconsistent and potentially biased annotations may have introduced noise into the training data.

When evaluating performance per class, speaking and coughing showed higher $F_{1}$ scores than the individual wet- and dry-swallowing classes. Wet and dry swallowing were frequently confused with each other, indicating that the present protocol and signal features do not reliably distinguish these two conditions. In contrast, aggregating wet and dry swallowing into a single swallowing class produced a substantially higher and more stable score (approximately $F_{1}=0.74$ in the reported analysis). This suggests that aggregated swallowing detection is a more appropriate primary target for the current feasibility study than classification of the bolus condition.

## 5. Conclusions

This controlled feasibility study demonstrates that two IMUs placed at the cricoid and thoracic/epigastric regions can detect prompted speaking, coughing, and swallowing events in ICU patients. Aggregated swallowing detection was more reliable than distinguishing wet from dry swallowing, while the thoracic accelerometer contributed particularly to cough detection. However, the study does not establish performance during spontaneous events or routine clinical activity.

The intended future application is the longitudinal estimation of event occurrence and frequency rather than immediate autonomous clinical decision-making. Before such an application can be considered, validation is required in larger and more clinically diverse cohorts, accounting for spontaneous events, nursing activities, posture changes, respiratory support conditions, sensor displacement, and longer wearing periods. Future studies should also evaluate false-alarm rates, conventional event-level detection metrics, and the clinical relationship between detected event patterns and relevant patient outcomes.

## Figures and Tables

**Table 1:** Baseline characteristics of the study population (SwaReCo001–SwaReCo029, n=29).

Characteristic	Value
Sex, *n* (%)	
Male	23 (79.3)
Female	6 (20.7)
Age (years), mean ± SD	68.4±11.5
Age (years), median [range]	72 [33–91]
Height (cm), mean ± SD	172.1±8.7
Height (cm), range	152–190
Weight (kg), mean ± SD	77.3±14.9
Weight (kg), range	47.2–118.7
BMI (kg/m^2^), mean ± SD	26.0±4.1
BMI (kg/m^2^), range	16.7–33.3
BMI category, *n* (%)	
Underweight (<18.5)	1 (3.4)
Normal (18.5–24.9)	9 (31.0)
Overweight (25.0–29.9)	14 (48.3)
Obese (≥30)	5 (17.2)
Recorded admission category, *n* (%)	
CABG via sternotomy	8 (27.6)
Aortic valve replacement via (mini)sternotomy, with or without CABG	7 (24.1)
Mitral valve replacement or repair via sternotomy, with or without CABG	2 (6.9)
Aortic valve replacement via right anterior thoracotomy	3 (10.3)
Mitral valve repair via port access	5 (17.2)
Non-surgical medical condition	4 (13.8)
ICU day at recording, *n* (%)	
Recordings occurred on day 1	1 (3.4%)
Recordings occurred on day 2	19 (65.5%)
Recordings occurred on day 3	6 (20.7%)
Recordings occurred on day 4	3 (10.3%)
Admission category, *n* (%)	
A	8 (27.6%)
B	7 (24.1%)
C	2 (6.9%)
D	3 (10.3%)
E	5 (17.2%)
F	4 (13.8%)

**Table 2:** Mean and standard deviation (std) of the recording length per recording type and the number of recordings (count). Recordings were started and stopped manually around the task.

Type	Mean (s)	Std (s)	Count
Coughing	15.41	6.28	28
Left turn	11.21	3.77	29
Right turn	9.48	4.37	29
Speech	16.45	4.78	29
Swallowing dry	23.79	6.65	29
Swallowing wet	30.07	8.69	30

**Table 3:** Number of retained features per signal channel after feature selection. The table specifies the final feature dimensionality used for the corresponding sensor configuration; counts are grouped by sensor location and signal modality.

Cricoid						Thorax		Total
ax	ay	az	gx	gy	C gz	a norm	g norm	
401	426	437	439	394	406	382	413	3298

**Table 4:** Overview of sensor configurations. Cricoid signals include tri-axial features; thorax signals are represented by their signal norms.

Configuration	Cricoid		Thorax	
	Acc.	Gyro	Acc.	Gyro
CA	**✓**	**✗**	**✗**	**✗**
CA+CG	**✓**	**✓**	**✗**	**✗**
CA+TA	**✓**	**✗**	**✓**	**✗**
CA+CG+TA+TG	**✓**	**✓**	**✓**	**✓**

**Table 5:** Number of samples per label for the 1 s and 1.5 s sliding window widths. The stride is fixed to 0.05 s.

Label	Samples	Events
Artifact	3111	121
Background	26,995	948
Coughing	3993	174
Speaking	7152	181
Swallowing dry	7776	149
Swallowing wet	9623	151

## Data Availability

The raw data supporting the conclusions of this article will be made available by the authors on request.

## Supplementary Materials

The following supporting information can be downloaded at https://www.mdpi.com/article/10.3390/s26217089/s1, Table S1: Predefined feature set (22 features) used in the sensitivity analysis. Features were computed per window [and per sensor channel]. Band energy was computed in five frequency bands, giving seven frequency features in total. References [25,26,27] are cited in Supplementary Materials.

## Abbreviations

The following abbreviations are used in this manuscript:

UES	upper esophageal sphincter
LES	lower esophageal sphincter
SS	spontaneous swallowing
VS	voluntary swallowing
SSR	spontaneous swallowing rate
ICU	intensive care unit
COPD	chronic obstructive pulmonary disease
ILD	interstitial lung disease
CF	cough frequency
VFSS	videofluoroscopic swallow study
FEES	fiber-optic endoscopic evaluation of swallowing
VAS	visual analog scale
CI	cough intensity
HRQoLQ	health-related quality of life questionnaires
HRCA	high-resolution cervical auscultation
MA	mechano-acoustic
sEMG	surface electromyography
ML	machine learning
AI	artificial intelligence
DL	deep learning
CNN	convolutional neural networks
DT	decision tree
IMU	inertial measurement unit
QoL	quality of life
BP	blood pressure
HR	heart rate
RR	respiratory rate
CO_2_	carbon dioxide
O_2_	oxygen
EWS	early warning score
LCQ	Leicester cough questionaire
IC	informed consent
PI	principal investigator
bpm	breaths per minute
LOPOCV	leave-one-patient-out cross-validation
LOSOCV	leave-one-subject-out cross-validation
TP	true positives
TN	true negatives
FN	false negatives
FP	false positives
ZCR	zero-crossing rate
CID	complex invariant distance
SHAP	SHapley Additive exPlanations
MAD	mean absolute deviation
