# Supplementary material for: Accelerometer- and Gyroscope-Based Sensor System for Detecting Swallowing, Speaking, and Coughing Events in ICU Patients
Source: Sensors (Basel). 2026 Nov 9;26(21):7089. doi: 10.3390/s26217089 (PMC13766363; doi:10.3390/s26217089)
Supplement: Supplementary file 1 [file sensors-26-07089-s001.zip › sensors-4553718-supplementary.pdf]

Supplementary Materials: Table S1

Table S1. Predefined feature set (22 features) used in the sensitivity analysis. Features were computed per window and per sensor channel. Band energy was computed in five frequency bands, giving seven frequency features in total.

Feature	Formula	Hyperparameters	Explanation
**Statistical Features**			
Mean	$\mu = \frac{1}{N}\sum_{i=1}^{N} x_i$	—	Average value over the window
Standard deviation	$\sigma = \sqrt{\frac{1}{N}\sum_{i=1}^{N}(x_i - \mu)^2}$	—	Measures spread around the mean
MAD	$\mathrm{MAD} = \frac{1}{N}\sum_{i=1}^{N}\lvert x_i - \mu\rvert$	—	Average absolute distance from the mean
Minimum	$\min(x)$	—	Smallest value in the window
Maximum	$\max(x)$	—	Largest value in the window
Skewness	$\frac{1}{N}\sum_{i=1}^{N}\left(\frac{x_i - \mu}{\sigma}\right)^3$	—	Measures asymmetry of the distribution
Kurtosis	$\frac{1}{N}\sum_{i=1}^{N}\left(\frac{x_i - \mu}{\sigma}\right)^4$	—	Measures peakedness or tail heaviness
Autocorrelation	$r(\tau) = \frac{\sum_{i=1}^{N}(x_i - \bar{x})\,(x_{i+\tau} - \bar{x})}{\sum_{i=1}^{N}(x_i - \bar{x})^2}$	$\tau = 2$	Correlation of the signal with a lagged version of itself
Number of Peaks	`find_peaks(x)` [25]	Default	Counts local maxima in the signal
Number of Valleys	`find_peaks(−x)` [25]	Default	Counts local minima in the signal
Zero Crossing Rate	$\frac{1}{N-1}\sum_{i=1}^{N} 1_{x_i x_{i+1}} < 0$	—	Counts sign changes in the signal
Linear Trend	Linear regression slope	—	Slope of the best-fit line over the window
Line Length	$\sum_{i=1}^{N-1}\lvert x_{i+1} - x_i\rvert$	—	Total absolute change in the signal; reflects activity
**Complexity Features**			
CID	$\mathrm{CID} = \sqrt{\sum_{i=1}^{N-1}(\Delta x_i)^2}$	—	Complexity estimate based on squared successive differences [26]
Lempel–Ziv Complexity	Lempel-Ziv complexity [27]	—	Measures the compressibility (randomness) of the signal
**Frequency Features**			

Dominant Frequency	$\text{ang max}_f \lvert X(f) \rvert$	—	Frequency with the highest energy in the spectrum
Spectral Entropy	$-\sum i\, p_i \log(p_i)$	—	Entropy of the normalized power spectrum
Band Energy	$\sum f \in [f_{min}, f_{max}] \lvert X(f) \rvert^2$	[0.1–1.5] Hz<br>[0.5–10] Hz<br>[1–20] Hz<br>[20–50] Hz<br>[50–100] Hz	Energy of the signal in the specified frequency bands
